# 检测Ⅰ期肺癌患者血清C反应蛋白的预后意义

**DOI:** 10.3779/j.issn.1009-3419.2011.05.04

**Published:** 2011-05-20

**Authors:** 烨 徐, 子明 李, 阳 申屠

**Affiliations:** 1 200336 上海，上海市长宁区中心医院胸外科 Department of Toracic Surgery, Shanghai Changning District Central Hospital, Shanghai 200336, China; 2 200300 上海，上海交通大学附属胸科医院/上海市肺部肿瘤临床医学中心 Aflliated Chest Hospital of Shanghai Jiaotong University/Shanghai Lung Tumor Clinical Medical Center, Shanghai 200300, China

**Keywords:** 肺肿瘤, Ⅰ期, C反应蛋白, 手术, 预后, Lung neoplasms, Stage Ⅰ, C-reactive protein, Operation, Prognosis

## Abstract

**背景与目的:**

急性炎症反应包括白细胞及急性期炎症蛋白的变化被较多研究证实是各种肿瘤包括非小细胞肺癌(non-small cell lung cancer), NSCLC的预后因素之一。本研究旨在观察Ⅰ期NSCLC患者术前C反应蛋白(C-reactive protein, CRP)水平与临床特征之间的关系及其预后意义。

**方法:**

应用胶乳免疫透射比浊法计算血清CRP含量回顾性收集2000年1月-2004年1月间上海市长宁区中心医院及上海市胸科医院共96例完整手术切除的Ⅰ期NSCLC患者资料, 采用*ANOVA*检验分析不同临床特征的患者中CRP水平是否存在差异。采用*Kaplan-Meier*曲线及*Logrank*检验进行生存分析和比较, 并采用*COX*多因素回归分析与生存相关的因素。

**结果:**

96例患者中CRP≤5 mg/L者66例。鳞癌(*P* < 0.001)、肿瘤直径 > 3 cm(*P* < 0.001)的患者CRP水平较高。多元线性回归分析提示肿瘤最大径与血清CRP水平相关(*β*=0.322, *P*=0.039)。CRP > 5 mg/L组患者的5年生存率低于CRP≤5 mg/L组(54.1% *vs* 78.2%, *P*=0.021)。*COX*分析表明CRP水平是影响Ⅰ期NSCLC患者总生存期的独立预后因素(*P*=0.023)。

**结论:**

Ⅰ期NSCLC患者术前CRP水平与肿瘤最大径呈正相关CRP > 5 mg/L组患者的总生存期低于CRP≤5 mg/L组可能是影响患者预后的不良因素。

## Abstract (en)

**Background and objective:**

The acute inflammatory, as manifest by alterations in white cell count and circulating concentrations of acute-phase proteins, C-reactive protein (CRP), and albumin, has been shown to be an independent prognostic factor in various cancers including non-small cell lung cancer (NSCLC). The aim of this study is to observe the relation between CRP level before operation and clinical characteristics of NSCLC stage Ⅰ, and its prognostic value.

**Methods:**

Data of ninety-six NSCLC stage Ⅰ cases under total operational excision from Jan 2000 to Jan 2004 were reviewed. The difference of CRP level in different characteristics was analyzed by *ANOVA*. Distributions and comparisons of survival according to CRP level were analyzed by *Kaplan-Meier* and *Log-rank* test. *COX* regression model was used to analyze prognostic factors of NSCLC.

**Results:**

Sixty-six cases had a CRP level of ≤5 mg/L. The level of CRP in patient with squamous cell carcinoma was remarkably higher than that with nonsquamous cell carcinoma (*P* < 0.001). The CRP level in tumor diameter > 3 cm was significantly higher than that in tumor diameter < 3 cm (*P* < 0.001). Multiple linear regression analysis suggested the maximum diameter of tumor was closely related to the level of serum CRP. The 5-year survival rate was lower in CRP > 5 mg/L group than that in CRP≤5 mg/L group (54.1% *vs* 78.2%, *P*=0.021). *COX* model found CRP level was an independent prognostic factor of stage Ⅰ NSCLC.

**Conclusion:**

The pre-operational CRP level of stage Ⅰ NSCLC is positively related to the maximum diameter of tumor. CRP level is probably a poor prognostic factor for stage Ⅰ NSCLC.

血清C反应蛋白（C-reactive protein, CRP）是一种炎症急性时相的反应蛋白，主要在白介素-6（interleukin-6, IL-6）的介导下由肝脏产生^[1]^。近年来有大量的研究表明CRP在结直肠癌^[2]^、食管癌^[3]^等人类肿瘤中可作为发病风险和影响预后的指标。最近又有研究^[4]^表明在晚期非小细胞肺癌（non-small cell lung cancer, NSCLC）中CRP水平也可作为患者的独立预后因素，但是否对早期手术的患者预后产生影响分析甚少。我们通过回顾性分析2000年1月-2004年1月上海市长宁区中心医院及上海市胸科医院接受手术治疗的Ⅰ期NSCLC患者的临床资料，旨在了解Ⅰ期NSCLC患者术前CRP水平与临床特征之间的关系以及对预后的影响。

## 1 资料与方法

### 1.1 临床资料

回顾性分析2000年1月-2004年1月上海市长宁区中心医院及上海市胸科医院接受手术治疗的共96例Ⅰ期NSCLC患者，平均年龄65岁（表 1）。术前分期工作包括：血液生化检查、胸部CT扫描、上腹部B超和CT、骨同位素扫描、纤维支气管镜检查、头颅CT或MRI检查。怀疑有远道转移的患者均排除在分析之外。所有患者均接受完整手术切除，支气管切断均为阴性。所有分期标准按照UICC第七版标准。

**1:** 纳入本研究患者的一般特征 General characteristics of patients included in this study

Characteristic	*n*
Sex	
Male	44
Female	52
Pathological type	
Squamous cell carcinoma	37
Non-squamous cell carcinoma	59
Smoking	
Non-smoker	55
Smoker	41
Level of C-reactive protein (CRP)	
CRP ≤ 5 mg/L	66
CRP > 5 mg/L	30

### 1.2 CRP水平检测

术前采外周静脉血5 mL，分离血清。采用胶乳免疫透射比浊法计算出CRP的含量。检测仪器为雅培公司生产的C8000型全自动生化分析仪。CRP正常范围：CRP≤5 mg/L。

### 1.3 统计学处理

患者的数据以SPSS 13.0统计软件进行统计处理，用*Kaplan-Meier*进行生存分析，*Log-rank*检验分析生存差异，建立*COX*回归分析了解患者的预后相关因素。*P* < 0.05为差异有统计学意义。

## 2 结果

### 2.1 临床特征与CRP水平的相关性

96例患者中CRP≤5 mg/L者66例。鳞癌（*P* < 0.001）、肿瘤直径 > 3 cm（*P* < 0.001）的患者CRP水平较高（表 2）。将年龄、性别、吸烟史、病理类型、肿瘤最大径与CRP水平进行多元线性回归分析，结果提示肿瘤最大径与血清CRP水平相关（*P*=0.039）。

**2:** 不同临床特征血清CRP水平 Comparison of the level of serum CRP with different clinical characteristics

Characteristic	CRP ≤ 5 mg/L (*n*=66)	CRP > 5 mg/L (*n*=30)	*P*
Age (year)			0.642
≤ 65	35	16	
> 65	31	14	
Sex			0.564
Female	31	19	
Male	35	11	
Smoking			0.264
Smoker	30	18	
Non-smoker	36	12	
Pathological type			< 0.001
Squamous cell carcinoma	12	26	
Non-squamous cell carcinoma	54	4	
Tumor max diameter (cm)			< 0.001
≤ 3	42	5	
> 3	24	25	

### 2.2 生存分析

96例患者中CRP≤5 mg/L组66例，CRP > 5 mg/L组30例。随访终止时，死亡27人，其中CRP≤5 mg/L组9例，CRP > 5 mg/L组18例。两组总生存曲线见图 1，提示CRP > 5 mg/L组5年生存率明显低于CRP≤5 mg/L组（54.1% *vs* 78.2%, *P*=0.021）。*COX*多因素分析结果见表 3，提示CRP水平是影响总生存期的独立因素（*P*=0.023）。

**1:**
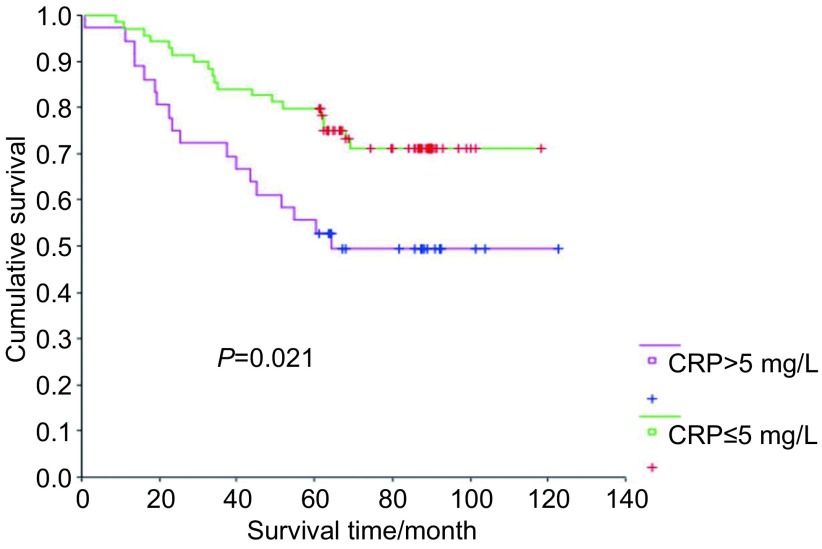
CRP > 5 mg/L组与CRP≤5 mg/L组患者的总生存期比较 *Kaplan-Meier* survival curve between CRP > 5 mg/L group and CRP≤5 mg/L group

**3:** 影响患者生存状况的多因素分析 Analysis survival condition affected by mutiple factors

	HR (95%CI)	*P*
Age (year)		0.220
≤ 65		
> 65	2.207 (1.391-3.256)	
Sex		0.230
Female		
Male	0.568 (0.354-1.107)	
Pathological type		0.055
Squamous cell carcinoma		
Non-squamous cell carcinoma	0.568 (0.354-1.107)	
Smoking		0.290
Non-smoker		
Smoker	1.468 (0.677-2.358)	
Tumor max diameter (cm)		0.078
≤ 3		
> 3	1.428 (0.987-2.549)	
Level of CRP		0.023
CRP≤5 mg/L		
CRP>5 mg/L	2.428 (0.746-2.221)	

## 3 讨论

参考文献CRP检测是一项有效的辅助检查指标，可用于感染的早期诊断和抗生素疗效的监测，被誉为炎症标志物而为人们所熟知。近年来研究^[5]^发现，血清中的CRP水平会随着炎症、损伤或肿瘤等各类疾病的变化而迅速增加或减少，因此可用于预测疾病的危险程度、跟踪病程、判断治疗效果和预后。研究^[6]^结果显示肺癌患者血清CRP水平的增高主要集中在有吸烟史的鳞癌患者。本研究发现男性、有吸烟史、鳞癌患者的CRP水平较高。考虑其原因是由于吸烟史的男性患者多为鳞癌，而鳞癌组织易坏死变性，且易出现阻塞性炎症，从而导致CRP水平的增高。

2009年出版的UICC第六版国际肺癌新分期标准中，肿瘤组织最大径成为影响患者分期以及预后的重要因素。而本研究结果发现CRP水平与肿瘤最大径正相关。这也预示着CRP水平对患者的预后也存在影响。通过对96例患者进行单因素分析，发现CRP > 5 mg/L组的患者其5年生存率明显低于CRP≤5 mg/L组（54.1% *vs* 78.2%, *P*=0.021）。有文献^[7]^指出肿瘤的进展以及预后与肿瘤以及自身的炎症反应有密切关系。有研究^[8]^表明大多数的实体上皮恶性肿瘤患者的体内存在炎症，有近半数的患者在诊断时可能存在有急性相反应，另外可能由于恶性肿瘤周边区巨噬细胞胞质或瘤细胞蛋白酶的释放，造成CRP合成的增加，最高可达300 mg/L。现研究^[9]^认为CRP的升高独立于肿瘤的分期之外可以作为许多类型的恶性肿瘤的预后因子。Kato等^[10]^研究发现治疗前浓度正常的CRP与增高的CRP患者的中位生存期存在明显差异(24. 9个月*vs* 3.7个月，*P* < 0.01)，提示CRP是晚期NSCLC生存预后的独立因素。国内学者潘建平等^[11]^对NSCLC患者的血清CRP水平与化疗疗效的相关性进行统计，其研究显示治疗有效组化疗后2周血清CRP浓度平均值下降了77.9%，而治疗无效组化疗前后血清CRP浓度平均值无明显变化。

本研究结果表明，肿瘤最大径是引起Ⅰ期NSCLC患者术前CRP水平增高的独立危险因素，CRP增高者5年生存率降低。提示对于接受手术治疗的Ⅰ期NSCLC患者，可以考虑通过术前血清CRP水平预测患者的临床特征并评价其预后，这无疑给临床提供了一个简便而又具有可重复性的方法。但本研究样本量较少，尚待前瞻性深入研究籍以进一步验证该指标的潜在价值。
